# Post-COVID-19 and Post-COVID-19 Vaccine Arthritis, Polymyalgia Rheumatica and Horton’s Arteritis: A Single-Center Assessment of Clinical, Serological, Genetic, and Ultrasonographic Biomarkers

**DOI:** 10.3390/jcm12247563

**Published:** 2023-12-08

**Authors:** Francesca Bandinelli, Mario Pagano, Maria Sole Vallecoccia

**Affiliations:** 1 Rheumatology Department, Usl Tuscany Center, San Giovanni di Dio Hospital, 50143 Florence, Italy; 2 National Research Council (CNR), Sesto Fiorentino, 50019 Florence, Italy; mario.pagano@cnr.it; 3 Anesthesia and Intensive Care Unit, Department of Emergency and Critical Care, Santa Maria Nuova Hospital, 50122 Florence, Italy; mariasole.vallecoccia@gmail.com

**Keywords:** arthritis, Polymyalgia Rheumatica, arteritis, COVID-19, COVID-19 vaccines

## Abstract

The potential role of the COVID-19 vaccine and infection to induce autoimmunity is currently underestimated despite the literature emphasizing arthralgia as a common adverse event. We aimed to study the impact of rheumatological complications post-COVID-19 (PC) and post-COVID-19 vaccine (PCV), comparing undifferentiated arthritis (UA) to Polymyalgia Rheumatica, Horton’s Arteritis (PMR-HA) and isolated arthritis to UA with “connective-like” accompanying symptoms. We retrospectively included 109 patients with at least 6 months of follow-up, analyzing serum biomarkers, joint ultrasound (US), lung HRCT, DLCO, and HLA haplotypes. There were 87 UA patients showing increased gastrointestinal and lung involvement (*p* = 0.021 and *p* = 0.012), higher anti-spike protein IgG levels (*p* = 0.003), and anti-SARS-CoV-2 IgG positivity (*p* = 0.003). Among them, 66 cases progressed to ACR-EULAR 2010 early arthritis after 3 months, whereas PMR-HA patients were more commonly PCV (81.8%, *p* = 0.008), demonstrating higher CRP (*p* = 0.007) and ESR (*p* = 0.006) levels, a lower rate of ANA positivity (*p* = 0.005), and a higher remission rate after six months (*p* = 0.050). In UA patients, the prevalent HLA was DRB1*11 and C*07 (36.8% and 42.1%). Serum calprotectin, interleukin-6, and C*07 (*p* = 0.021, 0.041, 0.018) seemed more specific for isolated UA. Conversely, “connective-like” arthritis showed poorer DLCO (*p* = 0.041) and more frequent US synovitis (*p* = 0.041). In conclusion, UA is a frequent common PC and PCV complication and may persist over time when compared to PMR-HA.

## 1. Introduction

The latest outbreak of COVID-19 (Coronavirus Disease 2019), attributed to the severe acute respiratory syndrome coronavirus-2 (SARS-CoV-2), has posed a significant global public health risk [1], resulting in approximately 7 million deaths all over the world [2]. 

COVID-19 can trigger varying levels of antibody responses [3], leading to the development of autoantibodies against human cell proteins in patients with severe disease [4]. While the reason for the variety of COVID-19 clinical consequences is not well understood, molecular mimicry occurring when unrelated proteins share regions of high molecular similarity, can provide an avenue for comprehension, allowing unexpected interactions to take place. COVID-19 and the Major Histocompatibility Complex (MHC) of human cells might encode similar peptide sequences, which allow the host to regard an infecting virus as “self” and forego an auto-immune response, disrupting self-tolerance and inducing cross-reactivity with host autoantigens [5,6]. The molecular mimicry between human proteins or other human pathogens and the spike antigenic protein of this virus, which is the most important reason for host-cell entry and the primary component in the vaccines against SARS-CoV-2, results in cross-reactive antibodies in response to SARS-CoV-2 infection or vaccination [7]. However, the reason for the variation in complex outcomes of cross-reactive antibodies is still under investigation, as it is hypothesized that genetic and environmental factors might contribute to an individual’s likelihood of developing an autoimmune response [8].

Recently, with the issuing of emergency use authorization, the commencement of vaccination against SARS-CoV-2 has been initiated to prevent severe disease that may necessitate hospitalization and reduce the risk of mortality [9]. 

Furthermore, while the safety and the benefits of creating herd immunity are widely supported by studies of the European (EULAR) and American (ACR) rheumatology societies and form part of a guidance statement for patients with rheumatic diseases [10], the rare but intricate new onset of autoimmune disorders [11,12] and a flare-up of existing rheumatic inflammatory diseases might arise after vaccination [13].

These occurrences could be linked to complex immunological mechanisms prompted by non-target or target antigens in vaccines, resulting in the undesirable excessive activation of immune reactions against self-antigens, with different immune responses and a large spectrum of severity [13,14]. The most common clinical signs of this vaccine-adverse event might include joint pain, inflammation and reddening around the joints, reduced mobility or stiffness, fatigue, and low-grade fever, which might be self-limiting in a large number of individuals [15]. Nevertheless, some case reports and series have demonstrated that new-onset arthritis might occur after vaccination among patients without previous autoimmune diseases. Ursini et al. [16] documented, in 66 patients, transient inflammatory musculoskeletal manifestations, including undifferentiated arthritis (UA) with oligo and polyarticular involvement and Polymyalgia Rheumatica. These predominantly occurred 11–13 days after COVID-19 vaccination (specifically after the BNT162b2 and AZD-1222 vaccines). 

Both the virus and vaccines can induce inflammatory arthritis [16,17,18], and various cases of rare systemic inflammatory disorders like vasculitis [19] adult, Still disease [20] and dermatomyositis [21] are reported.

It is important for clinicians to gain a better understanding of these conditions, which are often under or misdiagnosed. They are commonly perceived as less significant than idiopathic diseases, with a belief that they will run their natural course, resolving spontaneously and responding to steroids [19]. Otherwise, in the literature, the real esteem of their true incidence, treatment strategy, and genetic predisposition is still missing.

In this paper, we aim to study the impact of UA de novo cases in patients with post-COVID-19 (PC) and post-COVID-19 vaccine (PCV) rheumatological complications, evaluating its possible evolution in persistent early arthritis at follow-up and comparing their biomarkers to PC and PCV Polymyalgia Rheumatica and Horton’s arteritis (PMR-HA) cases of the same cohort. Finally, we studied the differences in these parameters and genetic haplotypes between UA with isolated arthritis and those patients presenting other accompanying “connective like” symptoms (e.g., Raynaud, sicca syndrome, myopathy).

## 2. Materials and Methods

### 2.1. Study Design

We conducted a retrospective analysis screening 177 Italian patients encountered in routine clinical practice, presenting with rheumatological complications of PC and PCV. These patients were referred to the early arthritis outpatient clinic of San Giovanni di Dio, either by general practitioners following regional flow chart guidelines or after hospitalization at the USL Tuscany Centre during the period between January 2021 (the beginning of the Italian COVID-19 vaccination period) and May 2023. In this study, we delineated PC and PCV rheumatological symptoms as new-onset alternative symptoms suggestive of UA, Polymyalgia Rheumatica, and Horton’s Arteritis (PMR-HA). These symptoms include girdle pain/stiffness, temporal headache, jaw claudication, and inflammatory peripheral joint and axial pain at rest with morning stiffness > 30 min [16] within four weeks between the first negative swab or COVID-19 vaccination. A routine swab was performed on all patients at the end of isolation to assess negativity following Italian Health Ministry legislation (law: 22 April 2021, no. 52, valid until repeal of law 10 August 2023, no. 105).

### 2.2. Participants

**Figure 1:**
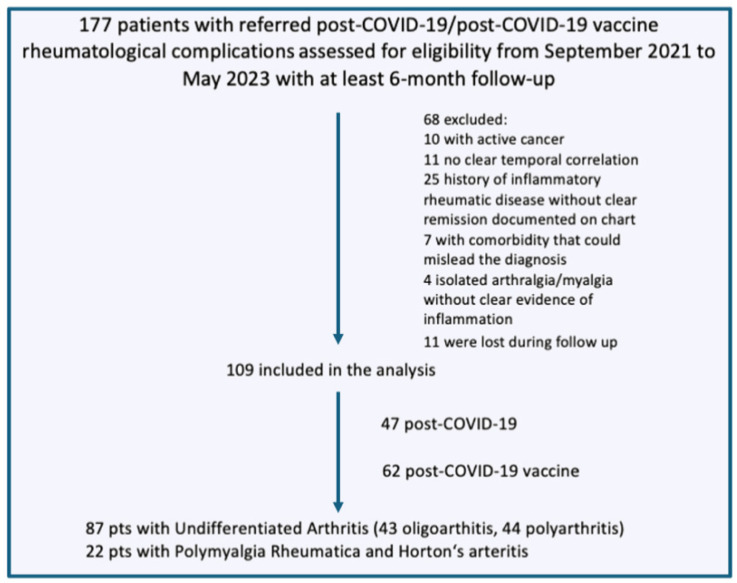
Flow chart of patient selection with exclusion criteria.

Data from 87 consecutive patients with UA (Figure 1) aged 18 years and older were gathered through the Argos electronic chart system of Tuscany Center. These patients exhibited a recent onset of arthritis symptoms occurring within 4 weeks after COVID-19 infection and COVID-19 vaccine [22], not previously treated with steroids or DMARDs for past arthritis. Exclusion criteria encompassed the presence of concomitant infections, tumors, neurological disorders (such as multiple sclerosis and cognitive impairment), fibromyalgia, and crystal-related arthritis, various shoulder conditions (e.g., bilateral rotator cuff syndrome and/or adhesive capsulitis, rotator cuff tear, glenohumeral osteoarthritis) or a follow-up shorter than six months. Additionally, patients solely complaining of non-specific long COVID-19 syndrome (isolated arthralgia/myalgia or vague and/or non-specific systemic symptoms such as fatigue or brain fog without clear inflammatory evidence) were also excluded (Figure 1). 

All UA patients were followed up at three months for the possible disease evolution of early arthritis, diagnosed using the EULAR/ACR 2010 criteria [23], and, at three and six months, the remission rate was evaluated. Privacy and informed consent (for anonymous analysis and publication of routine clinical data) were signed by each patient and saved in the Argos electronic chart of local health care service Tuscany center as per the Declaration of Helsinki on investigation of humans and according to the Tuscany Region Institutional Review Board resolution (No. 450) and Italian legislation (authorization No. 9, 12 December 2013). 

### 2.3. Measurements

We collected data regarding participants’ COVID-19 vaccination status, including their demographic, genetic, biochemical, and clinical attributes through patient records, face-to-face interviews, and clinical evaluations (oligoarticular or polyarticular involvement). Additionally, when required, we explored the possible association with other connective manifestations through capillaroscopy, salivary gland biopsy, and electromyography [EMG]. Furthermore, lung interstitial involvement was assessed through HRCT, analyzing elementary lesions: traction bronchiectasies, ground glass opacities, subpleuric fibrotic nodules, reticulation, honeycombing, fibrotic distortion, and fibrotic volume loss. In the presence of interstitial lung disease (ILD), the attending physician ordered the Diffusion Capacity of Carbon Monoxide (DLCO) and lung function test to be carried out. The severity and classification of DLCO reduction were defined as follows: normal DLCO: >75% of that predicted, up to 140%, mild: 60% to LLN (lower limit of normal), moderate: 40% to 60%, and severe: <40% [24]. We deemed a DLCO < 60% or a DLCO < 75% associated with a reduction in other restrictive parameter of the pulmonary function tests (forced vital capacity [FVC] < 80% of that predicted) to be significant [24,25].

Only the COVID-19 vaccination approved in Italy (BNT162b2, mRNA-1273, AZD1222, Ad26.COV2.S) was included in this study. The WHO (0–10) clinical progression scale was used to assess the severity of COVID-19 infection [26]. A “Paucisymptomatic” patient was defined as an individual with laboratory-confirmed SARS-CoV-2 infection with mild symptoms (WHO 2). No specific treatment guidelines were available for PC conditions at the time of this study.

UA patients’ biomarkers, remission rate, and treatments were compared to 22 subjects with new-onset (within 4 weeks since the infection or vaccine) PC and PCV PMR-HA, diagnosed using 2012 [27] and 2022 [28] ACR/EULAR provisional criteria, respectively, including Polymyalgia Rheumatica associated with Horton’s arteritis as part of the same comparison cohort.

Then, we divided UA into isolated and “connective like” arthritis subsets based on the presence of extra-articular clinical symptoms, and we compared serum biomarkers, the joint ultrasound, pulmonary involvement, and Human Leukocyte Antigen (HLA) genetic haplotypes. 

Venous blood specimens were collected after 12 h overnight fasting before beginning treatment and were analyzed at the Immunology and Genetic Laboratory of local health care service of Tuscany Centre (Florence and Prato, Italy, respectively).

The following blood tests were prescribed: erythrocyte sedimentation rate (ESR), C-reactive protein (CRP) (mg/dL), and Anti-Cellular Antibodies (ANA) tested via an indirect immunofluorescence assay using HEp-2 cells (Euroimmun, Lübeck, Germany) according to the ICAP classification [29], anti-citrullinated peptide antibodies (ACPA) (Anti-CCP EDIA™; Euro-Diagnostica, Malmö, Sweden), rheumatoid factor (RF) IgM (N Latex RF; Siemens AG, Munich, Germany), calprotectin (Calprest, Eurospital, Trieste, Italy), interleukin (IL)-6 (Human IL6 Instant Enzyme-linked Immunosorbent Assay), anti-SARS-CoV-2 IgM and IgG (nucleocapsid protein), antispike IgG (S1-RBD quantitative), immunophenotypes, and HLAB*, C*, DR*, DQ* haplotypes analyzed with inverse hybridization PCR (One Lambda, Canoga Park, CA, USA). Lastly, patients with arthritis-associated “connective like” symptoms, such as the Raynaud or sicca syndrome or myositis abnormalities at EMG, underwent investigation through specific blot for autoantibodies (Ab), and their parameters were compared to isolated arthritis.

Shoulders, wrists, hands, and other affected joints were investigated through a longitudinal and transverse ultrasound (US) examination performed by an Experienced Sonographer (B.F.) using a MyLab70 XVG machine (Esaote SpA, Genoa, Italy, multifrequency linear probe 12–15 MHz) with 750 Hz PRF and 53–55% dB gain for the Power Doppler (PD) setting. All images were saved in a digital archiving computer system. The US intra-observer agreement, tested on the same machine, was previously published [30], reporting good results (unweighted κ test = 0.90). The following echographic alterations were investigated [31]:

1. Active synovitis: echogenic, non-compressible, intra-articular synovial vascularization with a PD signal;
2. Active tenosynovitis or peri-tendinitis: hypoechoic thickened tissue with or without fluid within the flexor tendon sheath or around the extensor tendons, respectively, seen in two perpendicular planes, displaying a PD signal;
3. Pseudo-tenosynovitis (in hands): hypoechoic soft tissue surrounding the flexor tendon showing an intense PD signal;
4. Bone erosions: interruptions of the bone profile on two perpendicular scanning planes.

### 2.4. Statistical Analysis

The sample size was calculated on the basis of a similar recently published study [16]: 61 patients in the studied population were needed to achieve >80% power with an α level of 0.05 and beta 0.1. The Kolmogorov–Smirnov and Shapiro–Wilk tests were used to evaluate the distribution of variables. Data with a non-normal distribution were assessed using the Mann–Whitney U-test. Descriptive statistics were expressed as the median and interquartile range (IQR) for continuous parameters and the percentage for categorical variables, as appropriate.

Categorical data were compared among groups using the Chi-square test. The level of statistical significance was set at a *p*-value ≤ 0.05. All statistical analyses were performed using GraphPad Prism 8.0. The reporting of this study conforms to STROBE guidelines [32].

## 3. Results

A total of 177 PC and PCV patients with arthritis and PMR-HA and with recent onset (within the first three months from the beginning of symptoms) have been encountered during routine clinical practice at the early arthritis San Giovanni di Dio output clinic from September 2021 to May 2023. 

Of these, only 109 were included in the analysis (Figure 1): 47 of them developed symptoms after COVID-19 infection, 56 after an RNA-based vaccine, 2 after heterologous vaccination (RNA-based and viral vector vaccine), and 4 after the viral vector vaccine. The patients included did not receive any antiviral drug during COVID-19 infection or other biological treatment such tocilizumab or anti-Jak inhibitors. A specific analysis to track the variants of SARS-CoV-2 was not conducted.

Out of the 47 patients with PC, the majority (33/47) encountered a mildly symptomatic illness, classified on the WHO clinical progression scale as two. Additionally, 8/47 required domiciliary assistance (WHO 3), 1/47 was hospitalized without the need for oxygen therapy (WHO 4), and 2/47 received treatment with low-flow oxygen therapy (WHO 5). Moreover, 3/47 were managed with high-flow oxygen therapy or non-invasive ventilation (NIV) (WHO 6). At the onset, no patient tested positive for anti-SARS-CoV-2 IgM. 

Of these patients, 87 experienced an initial UA with oligoarticular onset in 43/87 (49.4%) and polyarticular onset in 44/87 (50.6%).

Initially, RF and ACPA were positive in 10/87 (11.5%) and 5/87 (5.7%) of individuals, respectively, and a joint ultrasound showed PD-active synovitis in 74/87 (85.1%), PD active proliferative tenosynovitis in 57/87 (65.5%), PD active pseudo-tenosynovitis in 53/87 (60.9%) and bone erosions in 5/87 (5.7%) individuals. At follow-up, a traditional hands and feet X-ray showed erosions in 17/87 (19.5%) of patients, and at 3 months of follow-up, 66/87 (75.9%) of patients met the ACR/EULAR 2010 criteria for early arthritis.

### 3.1. Comparison of PC and PCV Arthritis to Polymyalgia Rheumatica and Horton’s Arteritis

The UA patients’ clinical and therapeutic aspects and biomarkers, compared to 22 patients with PMR-HA, with a similar gender and age distribution, are shown in Table 1 and Table 2, respectively; the delay (days) between symptoms’ onset and COVID-19 recovery or vaccine administration were also not significantly different (UA mean 5 ± 0.8 SEM, 1–28 CI, vs. PMR-HA 2.8 ± 1.2, 1–25 CI). The clinical and therapeutic aspects and biomarkers of patients with UA, compared to 22 patients with PMR-HA, with similar gender and age distribution, are presented in Table 1 and Table 2, respectively. The delay (in days) between the onset of symptoms and recovery from COVID-19 or administration of the vaccine did not show significant differences (UA mean 5 ± 0.8 SEM, 1–28 CI, vs. PMR-HA 2.8 ± 1.2, 1–25 CI).

UA was more prevalent following COVID-19 infection (43/87 vs. 4/22, 49.4% vs. 18.2%, respectively, *p* = 0.008), whereas PMR and HA were more frequently reported after the COVID-19 vaccination (44/87 in UA vs. 18/22 in PMR-HA, 50.6% vs. 81.8%, *p* = 0.008). UA patients had a higher levels of anti-spike protein IgG (*p* = 0.003) and a higher percentage of anti-SARS-CoV-2 IgG positivity (*p* = 0.003). Girdle pain was observed in all cases (100%, *p* = 0.999), while the occurrence of headaches showed no significant difference between the UA and PMR-HA groups, with rates of 15/87 (17.2%) and 6/22 (27.2%), respectively (*p* = 0.363). Mandibular claudication was observed only in 4/22 (18.2%) of PMR-HA patients. On the other hand, interestingly, in comparison to PMR-HA, 17/87 UA reported a high incidence of initial gastrointestinal symptoms (17/87, 19.5% vs. 0%, *p* = 0.021), such as meteorism, abdominal pain, and diarrhea. Among these patients, only three had a significant elevation of fecal calprotectin (>100 mg/kg), which subsequently decreased during follow-up. Colon endoscopy was conducted in only one subject, revealing non-specific inflammation (oedema in all colon and rectum, and lympho-plasma cellular infiltration of lamina propria with micro-hemorrhages of the right colon). Furthermore, HRCT ILD with fibrotic signs was present only in 20/87 UA group (23%), none in PMR-HA and both in PCV (11/20) and PC (9/20) arthritis. In the UA PC group, among the 9 cases of ILD, 4 had a mild pauci-symptomatic (WHO 2) COVID-19 infection, 3 needed only domiciliary assistance (WHO 3), only 2 were hospitalized for a few days without invasive ventilation (WHO 6), 2 were mild smokers and none had a previously known relevant lung disorder.

**Figure 2:**
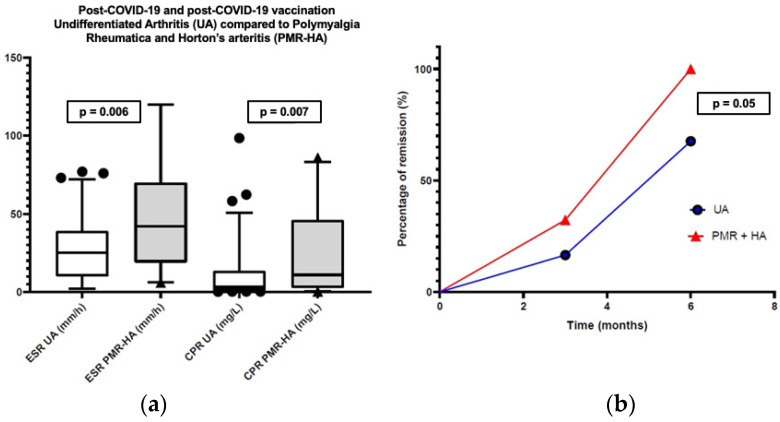
(**a**) Difference in acute-phase reactants (ESR and CRP) between post COVID-19 and post-vaccine arthritis (UA), Polymyalgia Rheumatica and Horton’s arteritis (PMR-HA); (**b**) A higher remission rate in patients with PMR-HA than in UA at three and at six months (significant at 6 months *p* = 0.05).

CRP and ESR (Figure 2) were more elevated (*p* = 0.007, *p* = 0.006), and the NK count was lower (*p* = 0.037) (Table 2 and Table S1) in PMR-HA; on the other hand, UA patients presented a higher percentage of ANA positivity (>1:160) (62/87 vs. 8/22, 71.3% vs. 36.4%, *p* = 0.005), while serum calprotectin and IL-6 were not different between the two groups.

As shown in Table 1, all patients in the PMR-HA group were treated with steroids, while the majority of patients in the UA group were treated with steroids (88.5%), hydroxychloroquine (67.8%), and vitamin D (67.8%).

After three months, there were no significant differences, but at six months of follow-up, remission was achieved in a larger proportion of PMR-HA patients (90.9%) compared to those with UA (59.8% *p* = 0.05) (Figure 2).

### 3.2. Comparison of PC and PCV Isolated Arthritis to “Connective Like” Arthritis

Among the 87 cases of PC and PCV arthritis, 30 initially presented mild “connective-like” (CL) symptoms following the negativization of infection and vaccination as follows: EMG motor unit potential (MUAPs) abnormalities (10/30, 33.0%), Raynaud (5/30, 16.7%), pitting scars (3/30, 0.1%), sicca syndrome (6/30, 20%) and antiphospholipid positivity (4/30, 13.3%) (see Supplementary File S1).

This group of CL arthritis patients was compared to those with isolated UA (Table 3 and Table S1 for immunophenotype). Of note, isolated arthritis patients had a higher positivity of serum calprotectin and IL-6 (28.1% vs. 6.7% *p* = 0.02, 25.6% vs. 6.7% *p* = 0.04). HRCT ILD was present in both groups (16.7% isolated UA vs. 26.3% connective-like UA, *p* = 0.186). 

The ILD elementary lesions on HRCT in PC and PCV are shown in Table 3. Traction bronchiectasis was the only considerably prevalent feature in isolated arthritis (24.5% UA vs. 13.3% CL, though not significantly different), with no other distinct signs indicative of a radiologic pattern (whether usual or non-specific ILD). Even the lung biopsy, conducted in only one case, did not provide discriminatory results.

CL patients showed lower DLCO levels (16.7% vs. 3.5%, *p* = 0.041) in functional respiratory tests and a higher rate of synovitis (90.0% vs. 70.2%, *p* = 0.041) in US compared to isolated UA patients (Table 3).

### 3.3. Comparison of HLA Haplotypes in PC Arthritis vs. PCV Arthritis and in Isolated Arthritis vs. Connective-like Arthritis

In all 87 UA patients, HLA was analyzed. The most common haplotypes were HLA DRB1*01 and DRB1*11 (16/87 and 32/87, 18.4% and 36.8%, associated only in 4 cases) and HLA C*06 and C*07 (16/87 and 30/87, 18.4% and 37.0%, associated only in 4 patients). Only in isolated arthritis the frequency of HLA C*07 was higher than in “connective like” arthritis (42.1 vs. 16.7%, *p* = 0.018), without any other difference between PC and PCV patients, as shown in Supplementary Table S2.

## 4. Discussion

Our study showed a higher incidence of UA following SARS-CoV-2 infection, characterized by a rapid and acute onset and progressing to early arthritis in 77% of patients within three months. Additionally, PMR-HA may emerge as a common adverse event of mRNA vaccines, with a notably elevated percentage of remission observed after six months.

In line with our study, a recent systematic review [17] of 190 case reports and case series from all parts of the world, published until September 2023, showed that the most common rheumatic diseases after COVID-19 vaccination were Polymyalgia Rheumatica, Horton’s arteritis, and arthritis. In addition, the majority (56.5%) of patients had previously received the BNT162b2 vaccine analogously to our study.

Furthermore, based on our data, the levels of ANA, anti-spike protein IgG, and anti-SARS-CoV-2 IgG (nucleocapsid protein) antibodies were found to be higher in the UA than in the PMR-HA group, indicating a more pronounced autoimmune activation in arthritis. Conversely, PMR-HA exhibited lower counts of natural killer (NK) cells compared to UA. 

Since the S1 spike subunit and nucleocapsid (N1) structural protein of SARS-CoV-2 are highly immunogenic and expressed abundantly during vaccine or infection response, the quantitative immunoassay of these proteins is widely employed to assess immunity after vaccination [33] or COVID-19 infection [34]. Even if their role in autoimmune diseases still remains a topic of controversy [35] and their presence might predict a robust immune response that protects against reinfection [36], in a large prospective study on post-COVID-19 patients, autoantibodies correlated with more severe disease and lower pulmonary lung function at follow-up. This was also evident in our UA patients when compared to PMR-HA [37].

In our study, UA evolved in early persistent arthritis during the follow-up period in 77% of patients, showed extra-articular involvement (lung and bowel), and needed more complex treatment with DMARDs and supportive medications. By contrast, PMR-HA appeared largely responsive to steroid monotherapy with a higher percentage of remission at six months. 

It is conceivable that the elevated levels of autoantibodies for SARS-CoV-2 proteins might be associated with a more pronounced and prolonged autoimmune response. However, the limited number of patients examined prevents us from offering a definitive interpretation of these data.

Furthermore, we assessed patients at three and six months since the onset of rheumatic symptoms, a duration that appeared sufficient for observing clinical variations in the UA and PMR-HA groups. This timeframe contrasts with a previous analogous study that reported an average follow-up of six weeks [16].

Nevertheless, the existing literature suggests that a visiting schedule within the first six months after acute COVID-19 may be deemed overly frequent, given that most known post-infective clinical variables are unlikely to undergo significant changes [38].

Therefore, it is plausible to hypothesize that rheumatological complications should be thoroughly investigated and more personalized in future post-COVID-19 follow-up schedules. Specific guidelines could be developed to mitigate excessive costs and resource utilization.

In our study, mandibular claudication and elevated ESR and CRP biomarkers seemed to be more specific for PMR-HA when compared to the UA group. Additionally, girdle pain was also a common feature in the UA group, as described previously by Ursini et al. [16]. Furthermore, PMR-HA patients showed a lower count of natural killer (NK) cells, the first fighters against an infected cell, in comparison to UA patients. Their function is also impaired in COVID-19 patients, leading to an inability to prevent the spread of this disease or destroy the infected cells [39]. Limited studies on subjects who received the BNT162b2 vaccine suggest that NK cell abnormalities may affect vaccine response [39]. While the literature reports only a reduction in CD8+ lymphocytes in PMR-HA before steroid treatment [40], such a reduction was not observed in our cohort. It is noteworthy that the occurrence of NK cell abnormalities in PC and PCV PMR-HA patients has not been reported until now.

ILD was present exclusively in UA (23%) patients and none in PMR-HA, with a predominance in “connective like” UA, whether in post-infective or in post-vaccine disease, suggesting a potential infective “trigger” based on the natural history of the disease.

Otherwise, it is challenging to discern a singular etiology and radiologic pattern of ILD in PC patients. In fact, we know that ILD is an important cause of morbidity and mortality in patients with rheumatic diseases [41] but is also central in the pathogenesis of COVID-19, causing pneumonia, even if it was rare in our cohort. 

Furthermore, gastrointestinal symptoms (meteorism, abdominal pain, and diarrhea) were also present in UA patients, with, in limited cases, a relevant increase in fecal calprotectin and histological evidence of non-specific inflammation (oedema in all colon and rectum and lympho-plasma cellular infiltration of the lamina propria with microhemorrhages in the right colon).

Several studies suggest that COVID-19 actively infects the cells of the gastrointestinal district, replicating itself in the epithelium of the small and large intestines.

This process triggers an excessive immunological reaction in the host, leading to the production of numerous cytokines via activated leukocytes [42]. Simultaneously, it is known that the intestinal microbiota plays a central role in the pathogenesis of rheumatic diseases [43].

A recent interesting Italian work evaluated the degree of bowel inflammation by measuring the fecal calprotectin (without employing endoscopy) in 16 symptomatic patients with a positive COVID-19 swab and radiological imaging of interstitial pneumonia, comparing them to 49 asymptomatic COVID-19-positive patients without interstitial pneumonia. The authors found an altered fecal calprotectin in 29.2% of all patients, with a higher frequency observed in cases of COVID-19 pneumonia (57.9%) compared to asymptomatic subjects (10.9%) [44].

A minor part of patients (34.4%) with arthritis presented mild and atypical connective symptoms at follow-up that evolved in a possible overlap only in isolated circumstances, as previously described in other series published [17,45]. In particular, there was only one severe myositis, which required prostaglandin, immunoglobulin, and mycophenolate treatments.

The comparison between these two aspects of the same disease, which has never been published, rendered intriguing cases of daily practice difficult to treat without specific guidelines yet recognized (File S1).

Interestingly, our analysis shows that “connective like” patients have poorer lung function (DLCO) and a more aggressive ultrasound pattern (active synovitis) than isolated arthritis. Moreover, we found that in isolated arthritis, calprotectin and IL-6 appeared to demonstrate greater specificity than in the connective pattern. These are recent biomarkers in rheumatic disease, either in early disease or in active disease during follow-up [30]. A recent study demonstrated, in post-COVID-19 intensive care patients, that serum calprotectin is higher than in the general hospital ward and correlated with lower respiratory DLCO [46]. Thus, even if the interpretation of our datum appears complex, the reason for this discrepancy might be the majority of pauci-symptomatic patients in our cohort.

In our patients with PC and PCV-undifferentiated arthritis, HLA DRB1*01-B1*011 and C*06–C*07, haplotypes usually correlated with early arthritis [30,47] and were adequately represented with a predominance of HLADRB1*11 and HLAC*07 (Table S2). HLADRB1*11 is one of the major gene determinants of the shared epitope disease susceptibility (together with HLA DRB1*01) and severity and mortality in rheumatoid arthritis [48,49,50]. It was recently studied, in the BNT162b2 vaccine, that HLA allele DRB1*11 seemed to have the largest predicted interaction with the protein product of the BNT162b2 mRNA vaccine containing immunogenic epitopes who trigger autoimmune phenomena in predisposed individuals through a molecular mimicry mechanism [51]. 

Finally, in our results, HLA C*07 resulted more specifically for isolated UA compared to “connective like” arthritis. The HLA-C gene is expressed in the maternal–fetal interface, playing an important first role in immunomodulation [52], and its importance in infectious disease is growing. Firstly, it was studied in human T-lymphotropic virus type 1 (HTLV-1) infections linked to a higher risk of myelopathy and, recently, was correlated with an increased mortality and susceptibility to severe acute respiratory SARS-CoV-2 syndrome by triggering an overactive immune response linked to the KIR mechanism of NK cells, mainly in particular cases of polymorphism.

## 5. Conclusions

Our findings highlight the importance of considering the potential autoimmune trigger of SARS-COV-2 infection and vaccination and developing therapeutic interventions to reduce PC and PCV complications. 

In this study, UA was a frequent PC and PCV complication, more persistent if compared to PMR-HA and with a complex treatment strategy beyond steroids. UA showed lower acute reactant levels and higher autoantibody levels (ANA—anti-spike protein IgG and anti-SARS-CoV-2 IgG) than PMR-HA. UA had more frequent lung involvement than PRM-HA, with lower DLCO in UA patients with “connective like” accompanying manifestations.

In PC and PCV patients, HLA DRB1*11 and C*07 epitopes seemed to be frequent in UA, while PMR-HA showed an immunophenotype (lower NK count) different from idiopathic PMR-HA.

These findings could shed light on the pathogenesis of rheumatological complications in COVID-19 and its vaccine. However, due to the limited number of patients in our cohort, this should be viewed as an explorative initiation for further research involving more comprehensive genetic and immunologic assessments. 

Still, in the years to come, identifying autoimmune potential and heterologous immunity through the identification of instances of molecular mimicry between the Spike protein, proteins from humans, or human pathogens could contribute to a deeper understanding of disease pathogenesis. 

This, in turn, has the potential to enhance therapeutic treatments and provide valuable insights for vaccine design in the context of SARS-CoV-2 infection.

## Figures and Tables

**Table 1:** Post-COVID-19 and post-COVID-19 vaccine arthritis clinical features and follow-up compared to Polymyalgia Rheumatica—Horton’s arteritis and their respective treatments.

	Arthritis (N = 87)	Polymyalgia Rheumatica—Horton’s Arteritis (N = 22)	*p*-Value
Age, years (median, IQR)	58.00 (47.00–74.00)	71.00 (54.75–79.00)	0.069
Female Gender (N, %)	54 (62.1%)	18 (81.8%)	0.129
Post COVID-19 (N, %)	43 (49.4%)	4 (18.2%)	**0.008**
Post COVID-19 vaccine (N, %)	44 (50.6%)	18 (81.8%)	**0.008**
Specific vaccine administered (N, %)	BNT162b2 35(79.6%) mRNA-1273 6 (13.6%) AZD1222 1 (2.3%) Heterologous 2 (4.6%)	BNT162b2 13 (72.2%) mRNA-1273 2 (11.1%) AZD1222 3 (16.7%) Heterologous 0 (0%)	0.524 0.070 0.999 0.999
Signs and symptoms			
Headache (N, %)	15 (17.2%)	6 (27.3%)	0.363
Mandibular claudication (N, %)	0 (0%)	4 (18.2%)	**0.001**
Gastro-intestinal symptoms (N, %)	17 (19.5%)	0 (0%)	**0.021**
HRCT ILD (N, %)	20 (23%)	0 (0%)	**0.010**
3-month remission (N, %)	23 (26.4%)	10 (45.4%)	0.118
6-month remission (N, %)	52 (59.8%)	20 (90.9%)	**0.050**
Treatments			
Hydroxychloroquin N (%)	59 (67.8%)	1 (4.5%)	**0.001**
Steroids N (%)	77 (88.5%)	22 (100%)	0.207
Methotrexate N (%)	10 (11.5%)	1 (4.55%)	0.457
Salazopyrin N (%)	32 (36.8%)	0 (0%)	0.001
Anti-TNF alpha N (%)	etanercept (1.1%)	0%	0.999
Pregabalin	36 (41.4%)	6 (27.3%)	0.327
L-carnitine and alpha-lipoic acid	11 (12.6%)	1 (4.5%)	0.453
Vitamin D	59 (67.8%)	13 (59.1%)	0.459
Prostaglandin E1	1 (1.2%)	0 (0%)	0.999
Immunoglobuline	1 (1.12%)	0 (0%)	0.999
Mycophenolate	3 (3.5%)	0 (0%)	0.999

Values are expressed as percentages (significance *p* of Chi square test) and the median and interquartile range (IQR) (*p* of Mann–Whitney test); HRCT: high-resolution CT; ILD: interstitial lung disease; TNF: tumor necrosis factor.

**Table 2:** Laboratory biomarkers of post-COVID-19 and post-COVID-19 vaccine arthritis and Polymyalgia Rheumatica.

	Arthritis (N = 87)	Polymyalgia Rheumatica and Horton’s Arteritis (N = 22)	*p*-Value
CRP (mg/dL) (median, IQR) cut-off < 0.5 mg/dL	0.32 (0.12–1.35)	1.28 (0.37–4.8)	**0.007**
ESR (mm/h) (median, IQR) cut-off: 0–25	25 (10–39)	43 (25–70)	**0.006**
RF positive (N, %)	10 (11.5%)	0 (0%)	0.208
ACPA positive (N, %)	5 (5.8%)	0 (0%)	0.581
RF and ACPA positive (N, %)	13 (14.8%)	0 (0%)	0.067
ANA positive (≥1:160) (N, %)	62 (71.3%)	8 (36.4%)	**0.005**
Serum calprotectin positive (N, %)	18 (20.7%)	4 (18.2%)	0.999
IL-6 positive (N, %)	16 (18.4%)	7 (31.8%)	0.240
Anti-spike protein antibody BAU Who/mL (median, IQR)	1632 (296–1632)	256 (83–776)	**0.003**
Anti-SARS-CoV-2 antibody IgG, AU/mL (median, IQR)	10.10 (0.50–41.05)	0.55 (0.20–6.85)	0.064
Anti SARS-CoV-2 antibody IgG, positive (N, %)	39 (44.8%)	2 (9.1%)	**0.003**
NK (cells/mcl) (median, IQR) cut-off: 200–400	233.9 (162–382.3)	141.5 (93.68–248.7)	**0.037**

Values are expressed as percentages (significance *p* of Chi square test) and the median and interquartile range (IQR) (*p* of Mann–Whitney test); abbreviations: CRP: C-reactive protein ESR: erythrocyte sedimentation rate IL-6: interleukin 6, RF: rheumatoid factor, ACPA: anti-citrullinated protein antibodies, NK: natural killer cells, ANA: antinuclear antibodies.

**Table 3:** Clinical, ultrasonography and laboratory biomarkers of the post-COVID-19 and post-COVID-19 vaccine isolated arthritis compared to “connective like” arthritis.

	“Connective Like” Arthritis (N = 30)	Isolated Arthritis (N = 57)	*p*-Value
Laboratory features			
CRP (mg/dL) (median, IQR) cut-off < 0.5 mg/dL	0.27 (0.13–0.64)	0.39 (0.12–2.57)	0.154
ESR (mm/h) (median, IQR) cut-off: 0–25	22.50 (10.50–35)	28.00 (9.50–44.50)	0.412
RF positive (N, %)	2 (6.7%)	8 (14.0%)	0.483
ACPA positive (N, %)	2 (6.7%)	3 (5.3%)	0.999
RF and ACPA positive (N, %)	4 (13.3%)	9 (15.8%)	0.999
ANA positive (≥1:160): N, %, median, IQR	23 (76.7%) 1:160 (1:160–1:320) Speckled: 21 (70.0%)	39 (68.4%) 1:160 (1:80–1:240) Speckled: 28 (48.1%)	0.471 0.133 0.073
Serum calprotectin positive (N, %)	2 (6.7%)	16 (28.1%)	**0.021**
IL-6 positive (N, %)	2 (6.7%)	14 (24.6%)	**0.041**
Anti-spike protein antibody, BAU Who/mL (median, IQR)	1316 (207–1632)	1632 (380–1632)	0.330
Anti SARS-CoV-2 antibody IgG, AU/mL (median, IQR)	19.57 (0.62–57.10)	4.10 (0.35–34.10)	0.075
Anti SARS-CoV-2 antibody IgG, positive (N, %)	21 (70.0%)	18 (31.6%)	**0.008**
NK (cells/mcl) Median (IQR) cut-off: 200–400	229.9 (150.0–364.8)	229.0 (161.8–380.4)	0.593
HRCT interstitial lung fibrosis (N, %)	5 (16.7%) Post infection 3 (10%) Post-vaccine 2 (6.6%) Subpleural fibrotic nodules 3 (10%) Traction bronchiectasis 4 (13.3%) Ground glass 3 (10%) Micro-honey combing 1 (3.3%) Reticulation 1 (3.3%)	15 (26.3%) Post infection 6 (10.5%) Post-vaccine 9 (15.8%) Subpleural nodules 6 (10.5%) Traction bronchiectasis 14 (24.5%) Ground glass 2 (3.5%) Micro-honey combing 1 (1.7%) Reticulation 2 (3.5%)	0.186
Decreased DLCO (N, %) (<60% or <75% with low FVC)	5 (16.7%) Post infection 3 (10%) Post-vaccine 2 (6.6%)	2 (3.5%) Post infection 1 (1.8%) Post-vaccine 1 (1.8%)	**0.041**
US synovitis (*)	27 (90.0%)	47 (70.2%)	**0.041**
US pseudo tenosynovitis (*)	21 (70.0%)	32 (55.2%)	0.251
US tenosynovitis (*)	17 (56.7%)	40 (70.2%)	0.240
US erosions	1 (3.3%)	4 (7.0%)	0.656

Values are expressed as percentages (significance *p* of Chi square test) and the median and interquartile range (IQR) (*p* of Mann–Whitney test); abbreviations: CRP: C-reactive protein ESR: erythrocyte sedimentation rate IL-6: interleukin 6, RF: rheumatoid factor, ACPA: Anti-citrullinated protein antibodies, HRTC: high-resolution computed tomography, DLCO: Diffusing Capacity Of The Lungs For Carbon Monoxide, FVC: forced vital capacity, NK: natural killer cells, ANA: antinuclear antibodies; US = ultrasound; (*) US was active at Power Doppler evaluation.

## Data Availability

Data are available if required.

## Supplementary Materials

The following supporting information can be downloaded at: https://www.mdpi.com/article/10.3390/jcm12247563/s1, File S1: “Connective like” patients’ description; Table S1: Difference of immunophenotype between arthritis and Polymyalgia Rheumatica-Horton’s arteritis patients; Table S2: Different HLA haplotypes in post COVID-19 compared to postvaccine arthritis and in isolated arthritis vs. “connective like” arthritis.
