# Supplementary material for: Post-COVID-19 and Post-COVID-19 Vaccine Arthritis, Polymyalgia Rheumatica and Horton’s Arteritis: A Single-Center Assessment of Clinical, Serological, Genetic, and Ultrasonographic Biomarkers
Source: J Clin Med. 2023 Dec 8;12(24):7563. doi: 10.3390/jcm12247563 (PMC10743539; doi:10.3390/jcm12247563)
Supplement: Supplementary file 1 [file jcm-12-07563-s001.zip › jcm-2731391-supplementary.pdf]

Supplementary material

FILE S1.

In undifferentiated patients with motor impairment, muscle pain and stiffness, electromyography (EMG) showed in 10/30 (33.3%) with motor unit potential (MUAPs) abnormalities (small, short, and polyphasic with early recruitment and spontaneous activity) in the lower limbs (mild in 9/10) and/or increased CPK (5/30, 16.7%) and aldolase parameters (9/30, 30%). Only five patients (three anti-Ro 52, one of which was anti-ku-associated, one anti-Mda, and one anti-HMGCR) were confirmed as positive for myositis autoantibodies almost in the second withdrawal at follow-up and did not undergo muscle biopsy, mostly due to the lack of severe manifestations.

Only one young patient with UA at onset and at follow-up evolved in a rapid increscent myositis with severe EMG protopathic muscle abnormalities and high aldolase and CPK levels and was immediately treated with intravenous prostaglandin E1, steroids, immunoglobulin, and successively with oral mycophenolate.

Patients with Raynaud phenomenon (5/30, 16.7 %) and pitting scars (3/30, 10 %) showed capillaroscopy multiple crossing and tortuous capillaries without a scleroderma pattern (mega capillaries or desertic areas) and were negative for anti-DNA Crithidia luciliae and scleroderma blot.

The persistent and complete sicca syndrome (6/30 patients, 20%), investigated first line with the BUT and Shirmer test and salivary glands US, encompassed a biopsy in the case of abnormalities.

In total, 5/30 patients (Shirmer test positive and US with moderate abnormalities) underwent a histological investigation (Chisholm and Mason classification, 0-4); 3 had a score >3 (2 III and 1 IV grade), and 2 were II grade.

In total, 4/30 reported positivity for antiphospholipid autoantibodies (Ab) at onset, confirmed in a second withdrawal, of which one had only a mild isolated anticardiolipin IgG positivity without thrombosis or miscarriage. The other three young patients with high anticardiolipin and antibeta2 GPI IgG and without other factors of risk during follow-up presented with nonspecific neurological symptoms (two, acute headache and one, acute major depression) and (2/3) showed hypertense signals of the white encephalic subcortex during an MRI or (1/3) cortical hypometabolism at PET which was treated only with cardio aspirin.

**Table S1 (supplementary)**. Immunophenotype	**Arthritis** (N= 87)	**Polymyalgia rheumatica** (N=22)	p-value
NK (cells/mcl) (median, IQR) cut-off: 200-400	**233.9 (162- 382.3)**	141.5 (93.68- 248.7)	**0.037**
CD3+CD4+ (cells/mcl) (median, IQR) cut-off: 650-1400	960.0 (702.3- 1271)	1016 (648.5- 2410)	0.424
CD3+ (cells/mcl) (median, IQR) cut-off: 1100-1700	1467 (1204-1827)	1763 (1064- 3368)	0.325
	**"Connective like" arthritis** (N=30)	**Isolated arthritis** (N=57)	p-value
NK (cells/mcl) Median (IQR) cut-off: 200-400	229.9 (150.0- 364.8)	229.0 (161.8- 380.4)	0.593
CD3+ (cells/mcl) Median (IQR) cut-off: 1100-1700	1482 (1299- 1752)	1460 (1192-1858)	0.554
CD3+CD4+(cells/mcl) Median (IQR) cut-off: 650-1400	973.0 (747.0- 1270)	958.0 (703.0- 1321)	0.969

Values are expressed in percentages (significance p of Chi square test) and the median and interquartile (IQR) (p of Mann–Whitney test); abbreviations: CRP: C-reactive protein, ESR: Erythrocyte Sedimentation Rate, IL-6: interleukin 6, RF: rheumatoid factor, ACPA: Anti-citrullinated protein antibodies, NK: natural killer cells, ANA: antinuclear antibodies.

**Table S2 (Supplementary)** HLA	**Post-COVID Arthritis** (N=43)	**Post-Vaccine Arthritis** (N=44)	p-value
DRB1*01 (N,%)	8 (18.6%)	8 (18.2%)	0.999
DRB1*11 (N,%)	17 (39.5%)	15 (34.1%)	0.825
DRB1*01-DRB1*11 association (N,%)	2 (4.7%)	2 (4.5%)	0.999
DRB1*03 (N,%)	9 (20.9%)	8 (18.2%)	0.999
DQB1*02 (N,%)	16 (37.2%)	11 (25.0%)	0.252
B*38 or B*44 (N,%)	5 (11.6%)	10 (22.7%)	0.257
C*06 or C*07 (N,%)	22 (51.2%)	20 (45.5%)	0.669
C*06(N,%)	10 (23.2%)	6 (13.6%)	0.280
C*07(N,%)	12 (27.9%)	18 (40.9%)	0.260
C06c07 association(N,%)	0 (0%)	4 (9%)	0.999
**HLA**	**“Connective like” arthritis** (N=30)	**Isolated arthritis** (N=57)	p-value
DRB1*01 (N,%)	5 (16.7%)	11 (19.3%)	0.999
DRB1*11 (N,%)	11 (36.7%)	21 (36.8%)	0.999
DRB1*01-DRB1*11 association (N,%)	2 (6.6%)	2 (3.5%)	0.25
DRB1*03 (N,%)	5 (16.7%)	12 (21.1%)	0.779
DQB1*02 (N,%)	11 (36.7%)	16 (28.1%)	0.335
B*38 or B*44 (N,%)	3 (10.0%)	12 (21.1%)	0.243
C*06 or C*07 (N,%)	12 (40.0%)	30 (52.6%)	0.367
C*06 (N,%)	7 (23.3%)	8 (14.0%)	0.371
C*07 (N,%)	5 (16.7%)	**24 (42.1%)**	**0.018**
C06-c07 association (N,%)	0	2 (3.5%)	0.999
